# Highly Flexible Fabrics/Epoxy Composites with Hybrid Carbon Nanofillers for Absorption-Dominated Electromagnetic Interference Shielding

**DOI:** 10.1007/s40820-022-00926-1

**Published:** 2022-09-17

**Authors:** Jong-Hoon Lee, Yoon-Sub Kim, Hea-Jin Ru, Seul-Yi Lee, Soo-Jin Park

**Affiliations:** 1 Department of Chemistry, Inha University, 100 Inharo, Incheon, 22212 Korea; 2 Korea Architecture Safety Testing and Research Institute (KASTI), 88 Gasan Digital 1-ro, Seoul, 08590 Korea

**Keywords:** Conductive polymer composites, Fracture toughness, Flexible composites, Absorption-dominated electromagnetic interference shielding

## Abstract

Epoxy-based nanocomposites can be ideal electromagnetic interference (EMI)-shielding materials owing to their lightness, chemical inertness, and mechanical durability. However, poor conductivity and brittleness of the epoxy resin are challenges for fast-growing portable and flexible EMI-shielding applications, such as smart wristband, medical cloth, aerospace, and military equipment. In this study, we explored hybrid nanofillers of single-walled carbon nanotubes (SWCNT)/reduced graphene oxide (rGO) as conductive inks and polyester fabrics (PFs) as a substrate for flexible EMI-shielding composites. The highest electrical conductivity and fracture toughness of the SWCNT/rGO/PF/epoxy composites were 30.2 S m^−1^ and 38.5 MPa m^1/2^, which are ~ 270 and 65% enhancement over those of the composites without SWCNTs, respectively. Excellent mechanical durability was demonstrated by stable electrical conductivity retention during 1000 cycles of bending test. An EMI-shielding effectiveness of ~ 41 dB in the X-band frequency of 8.2–12.4 GHz with a thickness of 0.6 mm was obtained with an EM absorption-dominant behavior over a 0.7 absorption coefficient. These results are attributed to the hierarchical architecture of the macroscale PF skeleton and nanoscale SWCNT/rGO networks, leading to superior EMI-shielding performance. We believe that this approach provides highly flexible and robust EMI-shielding composites for next-generation wearable electronic devices.

## Highlights

- Highly flexible carbon ink-loaded polyester fabric/epoxy composites with outstanding mechanical durability and absorption-dominant EMI-shielding characteristics are fabricated.
- The fracture toughness is ~ 38.5 MPa m^1/2^ and electrical conductivity is maintained after 1000 bending cycles.
- A superior electromagnetic interference *SE*/t of ~ 66.8 dB mm^–1^ was observed in the X-band frequency with over 0.7 absorption coefficient, related to the hierarchical structures composed of macro-scaled voids from the polyester nonwoven fabric skeleton and nano-scaled networks from SWCNTs/rGO.

## Graphical abstract

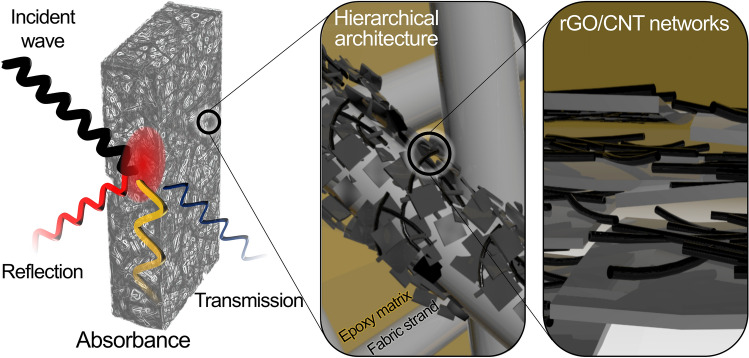

## Introduction

With the advent of the Internet of Everything (IoE) era, beyond the Internet of Things (IoT), has become highly linked to things (physical objects) as well as people, data, and processes. The IoE electronics for the human body, such as flexible sensors and health-monitoring systems, are expected to develop into lightweight, wearable, and human-friendly electronics [1, 2]. The IoE community is attempting to address the problem of electromagnetic (EM) waves emitted by electrical circuits or the electromagnetic interference (EMI) that occurs due to the operation of nearby devices. Such undesirable EM pollution could interfere with other delicate devices and cause the malfunction of interdigitated equipment. Human is likely to be at health risk, including headaches, insomnia, immune deficiency, and other diseases, through long-term EM wave exposure [3–5]. Therefore, there is great demand for the effective shielding of components to prevent the problems associated with EM waves [6, 7].

Normally, the reflection or absorption of EM waves is considered a promising strategy, and it can be obtained by using conductive materials, including metals, carbonaceous materials, and conductive polymer composites [8]. Conventional metal-based EMI-shielding materials, including aluminum, steel, and copper, have been explored owing to their high conductivity for efficient EMI-shielding. These are attributed to an impedance mismatch between free space and the shielding materials. Metals have exhibited reflection-dominant EMI-shielding performance through mobile charge carriers (electrons or holes) at the interface between media. However, the reflected EM wave causes secondary EM pollution, leading to undesirable interference in electronic devices and components. In this regard, EM reflection would be more severe in IoE communications due to a signal interruption via shorter wavelength and close module-to-module distance in the integrated system [9]. They also suffer from high density and processing costs, low chemical resistance, corrosion susceptibility, and poor flexibility, which restrict further EMI applications.

Absorption is one of the main mechanisms for EMI-shielding, mainly through materials with abundant electric and magnetic dipoles. This is considered an efficient way to reduce undesirable secondary EM emissions. Recently, carbonaceous material-loaded polymeric composites (CPCs) have become increasingly popular owing to their low density, superior corrosion resistance, ease of processability, and high tunability of the EMI-shielding performance in a wide range of frequencies, which is critical for the emerging class of applications in wearable electronics [10]. The multiple interfaces of CPCs provide scattering centers and interphases for efficiently attenuated reflections of the EM waves via absorption mechanism. Thus, CPCs have a lower surface reflection of EM waves than metals [11].

For a high EM absorption effectiveness, the CPCs require abundant electric dipoles with a high electrical conductivity that interacts with the EM waves. Carbonaceous materials, such as graphene [12], carbon black [13], carbon nanofibers [14], and carbon nanotubes (CNTs) [15], are an ideal choice as conductive fillers owing to their high electrical conductivity, low density, and superior physicochemical stability for the EMI-shielding applications. Several studies have used carbonaceous materials with EM compatibility for improving the data quality during IoE communications by attenuating unintentional EM waves [16–18]. Menon et al*.* studied the EMI-shielding performance using multi-walled carbon nanotubes (MWCNT)-based patch [19]. They mounted an EMI-shielding patch onto a Bluetooth module and demonstrated cutting off of incoming signals from a cellular phone. Jin et al*.* [20] fabricated cellulose nanocrystals (CNC)/reduced graphene oxide (rGO) films for the EMI-shielding materials. Hierarchically layered structures formed by interlaced CNC into rGO sheets facilitated multiple internal reflections and absorption of EM waves. It is demonstrated that the layered structure of the rGO nanosheets provided a high EMI-shielding performance.

A hybrid of single-walled CNTs (SWCNTs) and rGO has been used as conductive nanofillers to fabricate CPCs for EMI-shielding composites owing to their high electrical conductivity and superior ability to dissipate EM energy via conductive mismatch of filler–matrix interfaces [21, 22]. The addition of SWCNTs can increase the electrical conductivity because the SWCNTs fill the gaps between the graphene flakes and form a conductive network by bridging the neighboring graphene. The combination of SWCNT and rGO provides unique hierarchical structures by interlacing 1D CNT and 2D rGO hybrids. Such an architecture contributes porous structures and multiple interphases, thereby increasing the propagation paths of the permeated EM waves and promoting interfacial polarization loss for attenuating the EM waves [23, 24]. However, the agglomeration and restacking of the SWCNTs and rGO by themselves due to their electrostatic attraction and van der Waals forces limit the efficient EMI-shielding performance. In this regard, using a porous substrate to support the nanofillers of the SWCNTs and rGO can be a strategy to avoid agglomerations. Sheet-type substrate, such as nonwoven fabrics and porous paper, can be a good alternative to increase the degree of dispersion of rGO and the SWCNTs within the polymeric matrix [25, 26].

Additionally, many studies have focused on designing flexible films with mechanical durability for EMI-shielding [27, 28]. Zeng et al*.* [23] fabricated multi-walled CNT (MWCNT)/water-based polyurethane (WPU) films via a freeze-drying process to obtain EMI-shielding characteristics. They reported that the flexibility and mechanical endurance depended on the characteristics of the polymer substrates. The anisotropic porous structure of the MWCNT/WPU films facilitated an increase in the EMI-shielding properties. Wei et al*.* [29] prepared sulfonated graphene/polyacrylate films; the film exhibited high EM wave shielding of Bluetooth earphones with high flexibility and mechanical durability under 1000-cycle bending tests. Wang et al. [30] fabricated multi-functional EMI-shielding films using an MXene-sprayed nonwoven fabric. They reported that the mechanical strength of the films originated from aramid-based nonwoven fabrics. These composites had an EMI-shielding effectiveness (EMI-*SE*) of 35.7 dB under 8–12 GHz at the thickness of 1 mm.

These fabrics or paper-assisted CPCs, using thermoplastic polymers, exhibit high flexibility and ease of processability; however, some of their drawbacks of lower chemical stability and poor mechanical durability should be overcome. Epoxy, one of the thermosetting polymer, can be a promising alternative for robust EMI-shielding CPCs; however, they suffer from low flexibility due to their inherent brittleness [31, 32].

Here, we fabricated epoxy-based fabric nanocomposites using SWCNTs and rGO as conductive nanofillers. Polyester nonwoven fabrics (PFs) as a flexible substrate are explored to overcome the brittle nature of epoxy-based nanocomposites. In addition, the PFs play a role as a support for high dispersion of the nanofillers within a polymer matrix, facilitating to the EM wave attenuation due to the enhanced anisotropic direction of fabrics structure. The optimized carbon-loaded PF/epoxy composites exhibited superior electrical conductivity and fracture toughness of 30.2 S m^−1^ and 38.5 Mpa m^1/2^, respectively, with superior mechanical durability withstanding over 1000 bending cycles. The maximum EMI-*SE* was found to be 40.1 dB with the 0.6-mm-thick composites in the X-band frequency range of 8.2–12.4 GHz. The absorption-dominant behaviors were observed with an absorption coefficient of up to 0.7 with the presence of the SWCNTs. These results can be attributed to the hierarchical structures composed of macro-scaled voids from the PF skeleton and nano-scaled networks from SWCNT/rGO, which enhanced the absorption-dominant EMI-shielding and EMI-*SE*. We believe that this approach provides efficient EMI-shielding materials for next-generation wearable electronics with the advantage of ease of processability, high flexibility, and mechanical durability.

## Experimental Section

### Materials

The PFs (approximately 1 mm thickness) were obtained from Superbond Co., Korea. The SWCNTs (diameter of 5 nm and length of 10–20 µm) were obtained from Nanosolution Co., Korea. Graphite powder (particle size of ≤ 5 μm), sulfuric acid (H_2_SO_4_, 98%), phosphoric acid (H_3_PO_4_, 85%), potassium permanganate (KMnO_4_), hydrogen peroxide (H_2_O_2_, 30%), hydrogen chloride (HCl, 36%), and sodium dodecyl benzenesulfonate (SDBS) were purchased from Sigma-Aldrich Co., USA. The polymeric matrix system comprised an epoxy resin (diglycidyl ether of bisphenol A, YD-128) obtained from Kukdo Chemical Co., Korea, and a hardener (4,4'-diaminodiphenylmethane, DDM) obtained from Sigma-Aldrich Co., USA.

### Preparation of Reduced Graphene Oxide

The modified Hummers method was used to prepare graphene oxide from graphite through an oxidative exfoliation process [33, 34]. Here, 3 g of the graphite powder was stirred in a 9:1 mixture of H_2_SO_4_ (360 mL) and H_3_PO_4_ (40 mL) in an ice-water bath for 1 h. KMnO_4_, as an auxiliary oxidant, was slowly added to the mixture and stirred at 50 °C for 24 h. After cooling to room temperature (RT), sufficient quantities of H_2_O_2_ (5 wt%) and HCl (5 wt%) were gradually added to the mixture to remove the residues formed during the oxidation process. The mixture was washed repeatedly with deionized water until neutralized, and the unreacted graphite was excluded in this process. Lastly, GO was obtained by a membrane filter and dried at 50 °C in a vacuum oven. For rGO, GO was transferred to a tubular furnace and reduced at 900 °C for 2 h under a N_2_ atmosphere.

### Preparation of Hybrid Carbon Ink (SWCNT/rGO)

For the hybrid SWCNT/rGO inks, the SWCNTs were added to 100 mL of an rGO dispersion (1% by weight) as a function of SWCNT content. SDBS was added to the mixture containing SWCNT and rGO at a ratio of 1:99 (SDBS:SWCNT/rGO). The mixture was stirred at 25 °C for 8 h and then ultra-sonicated for 1 h until a homogeneous dispersion was observed [35, 36]. The carbon inks of SWCNT/rGO were labeled as “G” (no addition of SWCNT) and “S-$x$/G” ($x$ denotes the % by mass ratios of the hybrid nanofillers).

### Preparation of Hybrid Carbon Ink-Loaded Polyester Nonwoven Fabrics

We chose the PF as a matrix to fabricate flexible epoxy nanocomposites. A piece of PF was dipped in the hybrid SWCNT/rGO dispersion (carbon inks), whose preparation has been described in Sect. 2.2. The carbon inks were loaded onto the PF surfaces by an impregnation method in a vacuum oven at 80 °C for 5 h, and this process was repeated three times. The dried carbon ink-loaded PF was washed using deionized water several times to remove the surfactant until bubbles have not appeared. The carbon ink-loaded PF prepregs with different carbon inks were manufactured using a three-roll milling machine and then dried using a vacuum oven at 80 °C over 12 h. The prepared prepregs were denoted as G/PF, S-0.5/G/PF, S-1/G/PF, S-2/G/PF, and S-4/G/PF.

### Fabrication of the Hybrid Carbon Ink-Loaded PF/Epoxy Composites

**Fig. 1:**
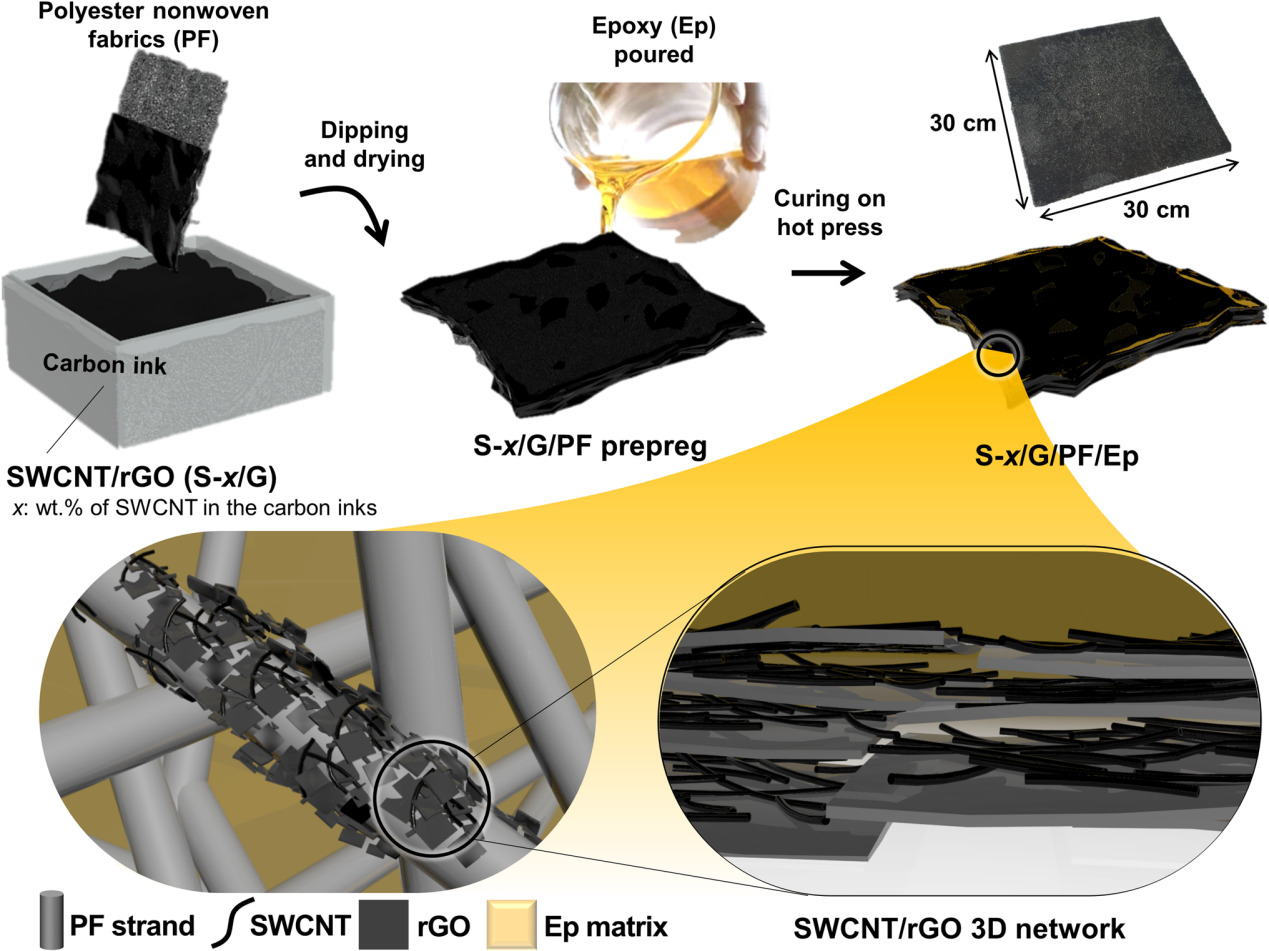
Schematic representation of the preparation process of the hybrid carbon ink-loaded polyester nonwoven fabric/epoxy composites (S-$x$/G/PF/Ep)

A mixture of the epoxy resin with DDM was prepared using a planetary mixer and then held in a vacuum oven at 60 °C for 30 min to completely remove the air bubbles. For the carbon ink-loaded PF/epoxy composites, the epoxy mixtures were poured on the prepregs of different carbon ink-loaded PFs. The composites were cured in the vacuum oven at 80 °C for 1 h and 120 °C for 1 h, followed by 180 °C for 2 h. This procedure was conducted with different carbon ink-loaded PF prepregs to obtain G/PF, S-0.5/G/PF, S-1/G/PF, S-2/G/PF, and S-4/G/PF, which are denoted as “G/PF/Ep,” “S-0.5/G/PF/Ep,” “S-1/G/PF/Ep,” “S-2/G/PF/Ep,” and “S-4/G/PF/Ep” (“PF/Ep” refers to polyester nonwoven fabric-impregnated epoxy composites). The overall preparation process for S-$x$/G/PF/Ep is presented in Fig. 1.

### Characterizations of the Carbon Ink-Loaded PF and Their Epoxy Composites

The morphologies of the prepreg specimens for the SWCNT/rGO-loaded PF (S-$x$/G/PF) were observed using high-resolution scanning electron microscopy (HR-SEM, SU-8010, Hitachi Co., Japan). The surface properties and microstructural properties of the prepregs before and after the addition of the SWCNTs were analyzed using a Fourier-transform infrared spectrophotometer (FT-IR, VERTEX 80 V, Bruker Co., USA) in the attenuated total reflectance (ATR) mode and Raman spectroscopy (Raman, LabRAm HR Evolution, HORIBA, Ltd., Japan), respectively. The structural phases of the prepregs were observed using X-ray diffraction (XRD, D2 PHASER, Bruker Co., Germany). The surface free energy was determined to characterize the interfacial properties of the composites by the sessile drop method with a contact angle (CA) goniometer (Phoenix 300 Plus, SEO Co., Korea). The wetting liquids used for the CA measurements were distilled water, ethylene glycol, and diiodomethane. The droplet volume of the wetting liquid was 5 µL, and the measurements were performed at RT. Three readings were taken from each sample, and the average values of the readings were used. The basic characteristics of the surface free energy of the liquids are listed in Table 1.

**Table 1:** Surface free energy components of the wetting liquids used in this study

Wetting liquids	$\gamma_{L}$ ^a^ (mJ m^−2^)	$\gamma_{L}^{L}$ ^b^ (mJ m^−2^)	$\gamma_{L}^{{{\text{SP}}}}$ ^c^ (mJ m^−2^)
Distilled water	72.8	21.8	51.0
Diiodomethane	50.8	50.4	0.4

^a^Surface free energy of probe liquid

^b^London dispersive component of probe liquid

^c^Specific polar component of probe liquid

### Mechanical Testing of the Hybrid Carbon Ink-Loaded PF/Epoxy Composites

The mechanical properties, including fracture toughness of the final resultant epoxy composites, were evaluated by a universal testing machine (LR-5 K plus, Lloyd instruments Co., UK) according to the ASTM D5045 standard. For each sample, five specimens were tested, and the average values were determined. The electrical conductivity of the composites was measured according to the ASTM D257 using a four-point probe electric resistivity tester (Mitsubishi Chemical Co., Japan) with platinum contacts. The specimens were 4 × 8 × 24 mm^3^ with a notch of 1.5 mm in depth in the middle, made with a band saw machine. The span-to-depth ratio and cross-head speed are 5:1 and 2 mm min^–1^, respectively.

The fracture toughness (${K}_{\mathrm{IC}}$) and strain energy release rates (${G}_{\mathrm{IC}}$) of the composites with varying SWCNT loading amounts can be calculated in terms of the stress and crack length using Eqs. (1)–(3), according to ASTM D5045 [37]:

1 $$K_{{{\text{IC}}}} = \frac{P}{B\sqrt W } \cdot f\left( {a/W} \right)$$

2 $$f\left( A \right) = \frac{{\left( {2 + A} \right)[0.886 + 4.64 \cdot A - 13.32 \cdot A^{2} + 14.72A^{3} - 5.6A^{4} ]}}{{\left( {1 - A} \right)^{3/2} }}$$

where *P* is the critical load, *W* is the specimen width, *B* is the specimen thickness, and *a* is the crack length of the specimen. In Eq. (2), *A* denotes ($a/W$):

3 $$G_{{{\text{IC}}}} = \frac{{\left( {1 - v^{2} } \right)K^{2}_{IC} }}{E}$$

where *E* is the Young’s modulus obtained from the fracture testing (MPa), and *v* is the Poisson’s ratio of the PFs, which was considered to be 0.34 [38, 39].

### EMI-shielding Effectiveness of the Hybrid Carbon Ink-Loaded PF/Epoxy Composites

The EMI-*SE* of the composites was evaluated according to ASTM D4935-89 (EM-6138, USA) in the 8.2–12.4 GHz frequency range using a vector network analyzer (GPC7-ES7, KEYCOM Co., Japan). The specimens were cut to dimensions of ø 1.2 cm × 0.6 mm for the EMI-*SE* tests. All specimens were measured at 23 ± 1 °C and a relative humidity of 50 ± 5%. The attenuation of the EMI can be calculated by Eq. (4):

4 $${\text{SE}} = 10\log \left( {\frac{{P_{T} }}{{P_{I} }}} \right)$$

where ${P}_{T}$ is the transmitted power, and ${P}_{I}$ is the incident power [40].

## Results and Discussion

### Characterization of the Hybrid Carbon Ink-Loaded PF

**Fig. 2:**
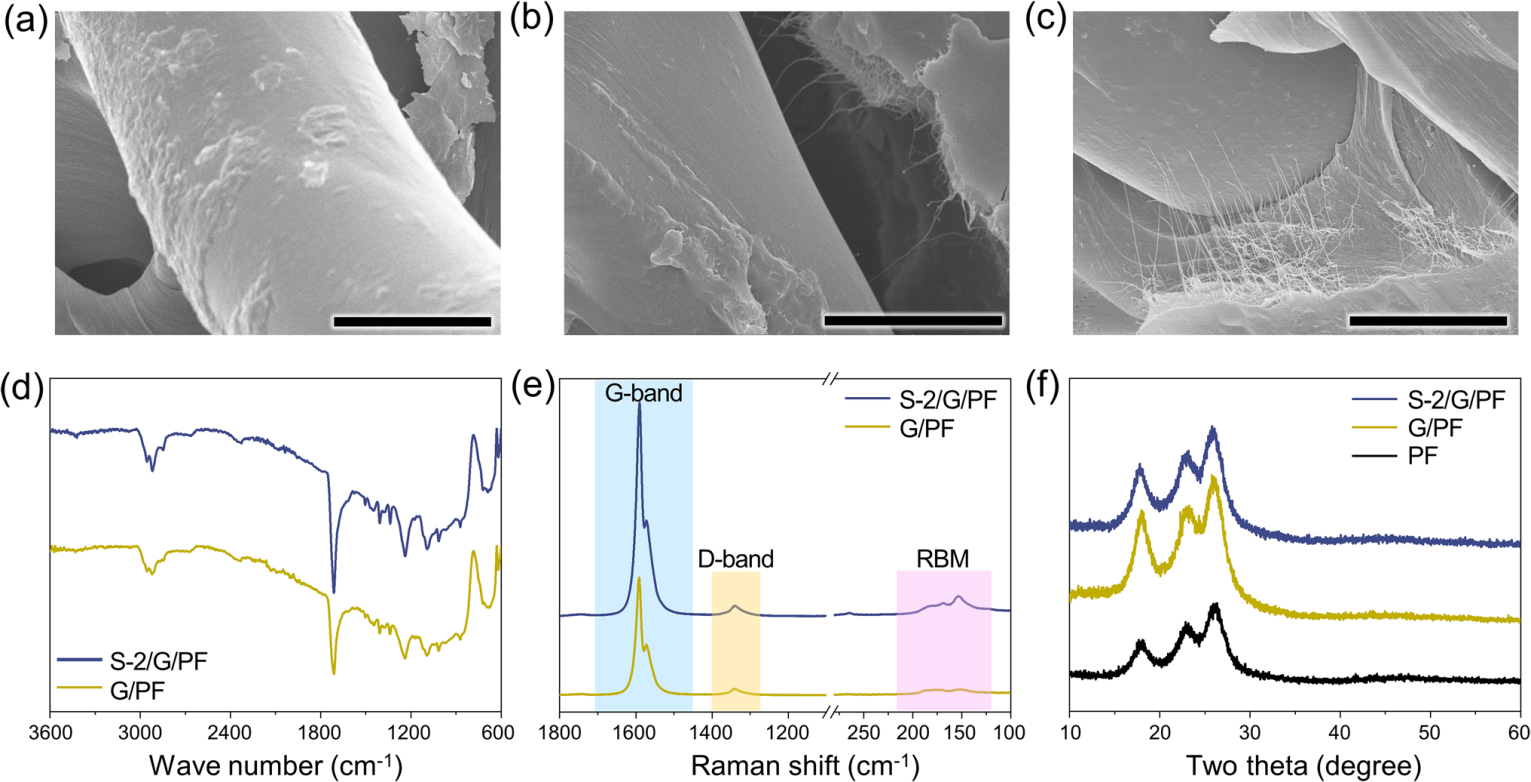
Characterization of the prepregs of the SWCNT/rGO-loaded polyester fabrics; SEM images of **a** G/PF, **b** S-2/G/PF, and **c** S-4/G/PF (scale bars: 10 µm), **d** FT-IR spectra, **e** FT-Raman spectra, and **f** XRD patterns

HR-SEM was used to investigate the morphological properties of the prepregs of the SWCNT/rGO-loaded PF samples. The SEM images were employed to observe the loading features of the rGO flakes and the hybrid SWCNT/rGO on the PF surfaces, as shown in Fig. 2a–c. Figure 2c shows densely packed hybrid SWCNT/rGO inks, and the agglomeration of the SWCNT bundles came out of the hybrid inks due to the excessive loading amount of the SWCNTs.

We used FT-IR spectroscopy to provide an insight into the chemical characteristics before and after loading the SWCNTs in the G/PF composites, as shown in Fig. 2d. The peak centered at 3040 cm^−1^ corresponds to *sp*^2^ hybridized C–H, while *sp*^3^ aliphatic C–H is observed at 2910 and 2850 cm^−1^. The prominent peak in S-2/G/PF was found at 1730 cm^−1^, which corresponds to the stretching vibrations of C = O, indicative of the presence of carboxylic acid and/or ester groups. The peaks at 1238 and 1093 cm^−1^ were assigned to the *sp*^2^ hybridized carbon atoms C–H and aromatic C–O stretching vibrations, respectively [41–43]. This implies that the *sp*^3^ carbon defects increased to some extent due to ultra-sonication during the hybridizing process of the SWCNT and rGO.

The Raman spectra were obtained to investigate the microstructural properties before and after loading of the SWCNT on the G/PF surfaces (Fig. 2e). The Raman spectra of the carbonaceous materials presented two main peaks corresponding to the G- and D-bands at approximately 1580 and 1350 cm^−1^, which can be attributed to the vibration of the* sp*^2^-bonded carbon atoms in a two-dimensional hexagonal carbon lattice and *sp*^3^ hybridized carbon defects and disorders in the graphitic lattice, respectively [44]. The prominent peaks of the G-band represent graphitic structures in both the G/PF and S-2/G/PF samples, while comparatively lower intensities of the peaks at 1350 cm^−1^ (D-bands) are exhibited. This means that the rGO and SWCNT were uniformly dispersed on the PF surface with few defects, resulting in comparatively larger intensity ratios of the G- and D-bands.

A typical radial breathing mode (RBM) in the Raman shift range of 100–200 cm^−1^ was distinctly observed in S-2/G/PF due to the presence of the SWCNTs. This characteristic peak of the RBM is attributed to the vibrational mode of the cylindrical shape that is not present in other graphitic structures [45]. The *I*_D_/*I*_G_ ratios, which is the ratio of *sp*^3^/*sp*^2^ of the carbonaceous materials, are 0.06 for S-2/G/PF and 0.37 for G/PF, respectively. The higher *I*_D_/*I*_G_ value of S-2/G/PF clearly corresponds to the presence of the SWCNTs, whereas the lower *I*_D_/*I*_G_ value of G/PF can be attributed to lower* sp*^2^ species of rGO. The structural preservation of the PF skeletal framework after the loading of the carbon inks is confirmed by maintaining peaks at 2θ = 18, 23, and 26° in the XRD spectra (Fig. 2f) [46].

### Determination of the Interfacial Properties of the Carbon Ink-Loaded PF/Epoxy Composites

The surface free energy can directly affect the interfacial dynamic forces, for example, the adsorption (gas–solid), wettability (liquid–solid), adhesion (solid–solid), and morphology of the components of the composites. It is well recognized that the interfacial properties of the composites are related to their structural integrity at both microscopic and macroscopic scales, thereby affecting most of the main properties in the composites, including mechanical behaviors [47].

The surface free energy (wettability) of the composites can be calculated from the CA method between a liquid and solid. Herein, the surface wettability was calculated between the liquid and the nanocomposites. According to Fowkes [48], Kaelble [49], and Owens and Wendt [50], the surface wettability can be divided into two components:

5 $$\gamma = \gamma^{L} + \gamma^{SP}$$

where $\gamma$ is the surface free energy, $\gamma^{L}$ is the dispersion component (Lifshitz–van der Waals interactions encompassing London forces), and $\gamma^{{{\text{SP}}}}$ is the specific polar component (Debye-inductive polarization, Keesom forces, and hydrogen bonding).

According to Fowkes’ suggestion [48], the surface free energy of a solid material can be examined by the CA using Eqs. (6) and (7):

6 $$\gamma_{L} \left( {1 + \cos \theta } \right) = 2\left( {\sqrt {\gamma_{S}^{L} \cdot \gamma_{L}^{L} } + \sqrt {\gamma_{S}^{{{\text{sp}}}} \cdot \gamma_{L}^{{{\text{sp}}}} } } \right)$$

7 $$\frac{{\gamma_{L} \left( {1 + \cos \theta } \right)}}{{2\sqrt {\gamma_{L}^{L} } }} = \sqrt {\gamma_{S}^{{{\text{sp}}}} } \left( {\frac{{\sqrt {\gamma_{L}^{{{\text{sp}}}} } }}{{\sqrt {\gamma_{L}^{L} } }}} \right) + \sqrt {\gamma_{S}^{L} }$$

where $\theta$ is the liquid droplet CA, and the subscripts $L$ and $S$ denote liquid and solid, respectively. The CAs were determined using the three wetting liquids, and Eqs. (5) and (6) were used to calculate the surface free energy.

The CAs of the carbon ink-loaded PF/epoxy composites were determined with different liquids, as listed in Table 2. Distilled water and diiodomethane were used as highly polar and dispersive liquids to estimate the polar and dispersive characteristics of the composites, respectively. The polar characteristics of the PF/epoxy composites increased with the increasing SWCNT content up to 2% by weight and then decreased. The data showed that the addition of the SWCNTs plays an important role in the surface interactions of the composites [51]. The larger interfacial areas of the 3D hierarchical architecture obtained by the interwoven SWCNT and rGO can contribute to an increase in the interfacial interactions [51, 52]. The excessive addition of the SWCNT appeared inefficient in increasing the surface interaction due to their agglomeration. This well corresponds with the SEM image shown in Fig. 2c. A contrasting tendency was observed in the case of diiodomethane.

**Table 2:** Contact angles of the carbon ink-loaded PF/epoxy composites (unit: °)

	Contact angles ($\theta$)	
	Distilled water	Diiodomethane
G/PF/Ep	78.2 ± 0.3	42.1 ± 0.2
S-0.5/G/PF/Ep	64.2 ± 0.4	43.4 ± 0.3
S-1/G/PF/Ep	61.8 ± 0.4	42.3 ± 0.2
S-2/G/PF/Ep	61.6 ± 0.3	41.6 ± 0.4
S-4/G/PF/Ep	63.7 ± 0.2	42.6 ± 0.3

**Fig. 3:**
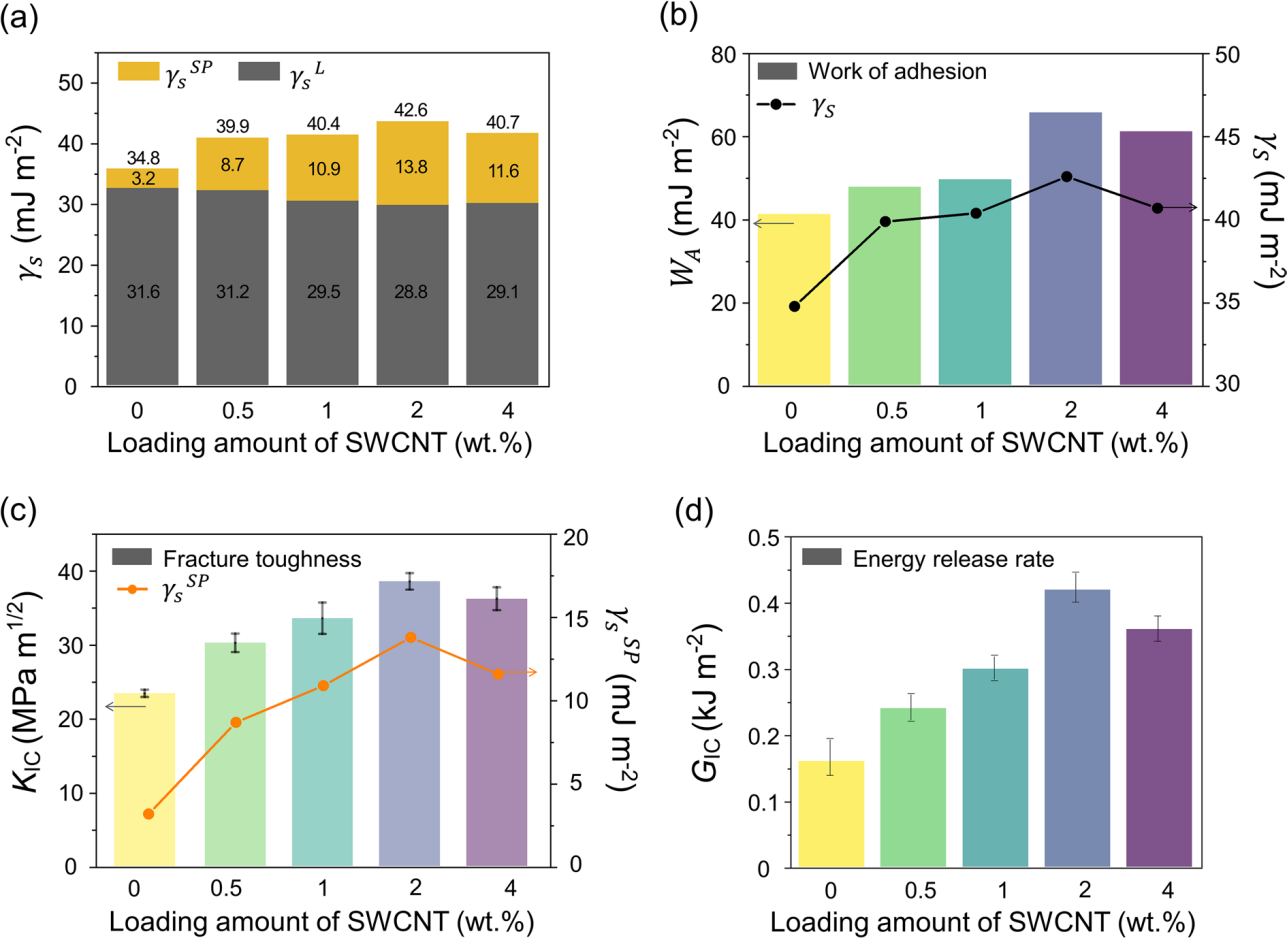
Interfacial and mechanical behaviors of the carbon ink-loaded PF/epoxy composites: **a** surface free energy (${\gamma }_{\mathrm{s}}$) with specific polar (${\gamma }_{S}^{\mathrm{sp}}$) and dispersion (${\gamma }_{S}^{L}$) components, **b** work of adhesion (${W}_{\mathrm{A}}$), **c** plane strain fracture toughness (${K}_{\mathrm{IC}}$), and **d** strain energy release rates (${G}_{\mathrm{IC}}$)

To further explore the surface free energy, the specific polar ($\gamma_{S}^{{{\text{sp}}}}$) and dispersion ($\gamma_{S}^{L}$) components of the composites are shown in Fig. 3a. The experimental data of the entire surface free energy in the carbon ink-loaded PF/epoxy composites significantly increased from ~ 40 to ~ 43 mJ m^–2^ with the increase in the SWCNT content compared to that of G/PF/Ep (34.8 mJ m^–2^) without adding SWCNTs. The increase in the $\gamma_{S}^{{{\text{sp}}}}$ components is shown to be predominant, while the $\gamma_{S}^{L}$ components were not significantly changed. The $\gamma_{S}^{{{\text{sp}}}}$ component of S-2/G/PF/Ep was 13.8 mJ m^–2^, which is four times higher than that of G/PF/Ep (3.2 mJ m^–2^), which indicates that the greater interfacial interactions of the carbon ink-loaded PF/epoxy composites can be attributed to newly formed interfacial areas that arise from the 3D hierarchical structures of the hybrid carbon fillers.

The work of adhesion or interfacial adhesion energy ($$W_{A} )$$ is an effective tool for evaluating the affinity between liquid probes and solid surfaces representing surface characteristics. The values of $$W_{A}$$ can be divided by the dispersion and specific bonding forces as follows:

8 $$W_{A} = \gamma_{L} \left( {1 + \cos \theta } \right)$$

9 $$= 2\left( {\sqrt {\gamma_{S}^{L} \cdot \gamma_{L}^{L} } + \sqrt {\gamma_{S}^{{{\text{sp}}}} \cdot \gamma_{L}^{{{\text{sp}}}} } } \right).$$

Figure 3b shows that the $W_{A}$ values of the carbon ink-loaded PF/epoxy composites increased with an increase in the SWCNT content up to 2% by weight and then decreased slightly. Among them, the highest $W_{A}$ was found to be ~ 66 mJ m^–2^ in S-2/G/PF/Ep, which is a 60% increase compared to that of G/PF/Ep. This indicates good linearity of the relationship between the surface free energy and $W_{A}$, demonstrating the enhanced interfacial properties.

To further explore the interfacial properties of the composites studied, plane strain fracture toughness ($K_{{{\text{IC}}}}$) and strain energy release rates ($G_{{{\text{IC}}}}$) were obtained, as shown in Fig. 3c, d. Our data demonstrated a good correlation between the $K_{{{\text{IC}}}}$ and $\gamma_{S}^{{{\text{sp}}}}$ components. The $K_{{{\text{IC}}}}$ value of S-2/G/PF/Ep was ~ 39 ${\text{MPa m}}^{1/2}$, which is ~ 67% higher than that of G/PF/Ep (~ 23 ${\text{MPa m}}^{1/2}$). This means that the specific polar component closely governs the fracture toughness of the composites. For the $G_{{{\text{IC}}}}$ values of the composites, the highest $G_{{{\text{IC}}}}$ value of S-2/G/PF/Ep was 0.42 kJ m^–2^, which is an increase of ~ 160% compared to that of G/PF/Ep. These results substantiate that the addition of SWCNT/rGO led to an improvement in the interfacial adhesion. However, the compatibilities in S-4/G/PF/Ep decreased due to the excessive SWCNT loading amount, resulting from the critical agglomeration of the SWCNTs in the hybrid system. In addition, the agglomeration of the fillers resulted in the decrease in the mechanical properties.

### Electrical Properties of the Hybrid Carbon Ink-Loaded PF/Epoxy Composites

**Fig. 4:**
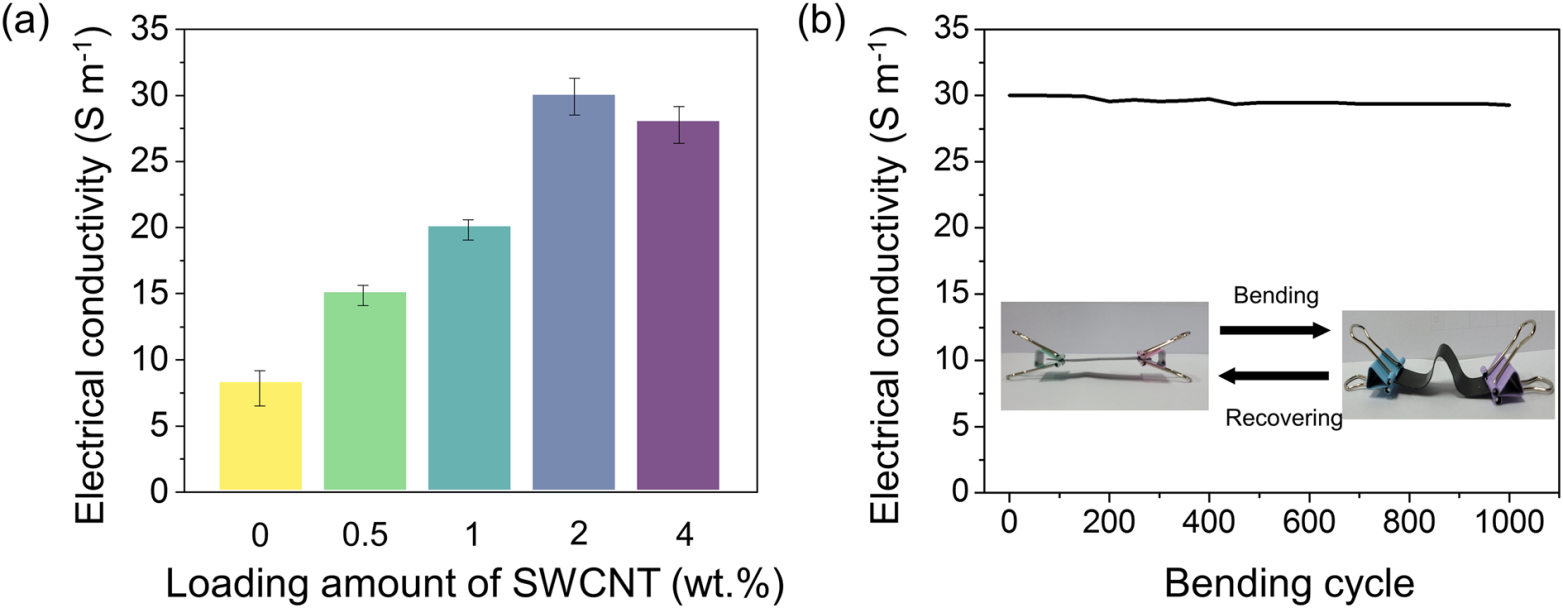
**a** Electrical conductivity of the hybrid carbon ink-loaded PF/epoxy composites as a function of the SWCNT content and **b** the durability test of S-2/G/PF/Ep during the mechanical deformation of 1000 cycles

The electrical conductivity of the carbon ink-loaded PF/epoxy composites as a function of the SWCNT content is shown in Fig. 4a. G/PF/Ep exhibited the lowest electrical conductivity of 8.2 S m^−1^, whereas S-2/G/PF/Ep exhibited the highest value of 30.2 S m^−1^. The obtained data revealed that the presence of SWCNTs plays an imperative role in improving the electrical conductivity of the composites. Interestingly, the electrical conductivities of the composites exhibited a same trend with the change of the interfacial properties, $K_{{{\text{IC}}}}$ and $G_{{{\text{IC}}}}$, depending on the SWCNT loading contents. The excessive loading amount of the SWCNTs could be undesirable for an efficient conductive network of the composites due to the lower interfacial properties, as discussed in Sect. 3.2 [53, 54].

Since mechanical durability is crucial for practical EMI applications, the bending test of the composites was performed to observe a constancy in the electrical conductivity when subjected to mechanical deformations over 1000 times (Fig. 4b). The data showed negligible deterioration during the mechanical deformation. This means that the optimized loading of the SWCNTs provided a well-developed percolation network in the nanocomposites. Further, the superior interfacial interactions among the rGO/SWCNT, epoxy, and PF substrate could lead to high mechanical durability [55].

### EMI-shielding Behaviors of the Hybrid Carbon Ink-Loaded PF/Epoxy Composites

**Fig. 5:**
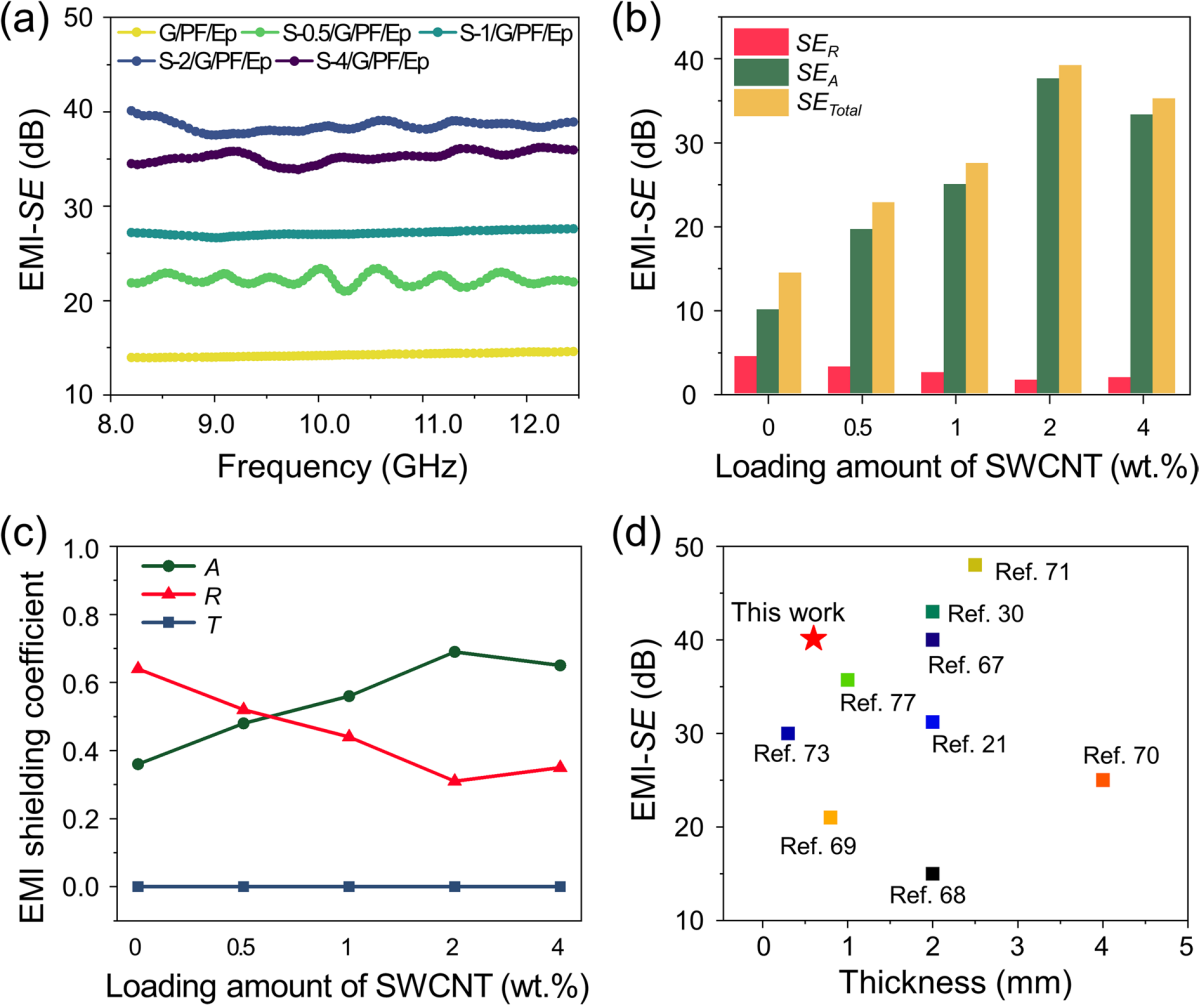
Electromagnetic interference shielding effectiveness (EMI-*SE*) of the S-*x*/G/PF/Ep as a function of the SWCNT content at the frequency of the X-band: **a** EMI-*SE*, **b** *SE*_*R*_, *SE*_*A*_, and *SE*_Total_ at a frequency of 8.2 GHz, **c** EMI-shielding coefficient of the composites at 8.2 GHz, and **d** comparison of the EMI-*SE* with different EMI-shielding materials

The EMI-*SE* is an important indicator for the quantitative shielding performance. The higher the EMI-*SE*, the lesser the EM waves transmitted through the shielding materials. The EMI-shielding profiles of the carbon ink-loaded PF/epoxy composites in the X-band frequency of 8–12.4 GHz are shown in Fig. 5a. The data revealed that the EMI-*SE* of the samples improved with increasing SWCNT content but sharply decreased in S-4/G/PF/Ep owing to the agglomeration of the SWCNTs in the hybrid system. The highest EMI-*SE* was found to be 40.1 dB in S-2/G/PF/Ep, which is an improvement of ~ 185% compared to that of G/PF/Ep. These data imply that the EMI-*SE* is highly governed by the intrinsic electrical conductivity and the degree of dispersion of the nanofillers, which has strongly influenced the interfacial interactions of the composites.

According to the *Schelkunoff* theory, the EMI-*SE* can be defined as the sum of three contributions—absorption (${SE}_{A}$), reflection (${SE}_{R}$), and multiple reflections (${SE}_{M}$) [56, 57], through the following Eqs. (10)–(13):

10 $${\text{SE}}_{{\text{Total }}} = 10\log \left( {\frac{{P_{i} }}{{P_{t} }}} \right) = 20\log \left( {\frac{{E_{i} }}{{E_{t} }}} \right) = 20\log \left( {\frac{{H_{i} }}{{H_{t} }}} \right)$$

11 $$= {\text{SE}}_{A} + {\text{SE}}_{R} + {\text{SE}}_{M}$$

12 $${\text{SE}}_{R} = - 10\log (1 - S_{11}^{2} )$$

13 $${\text{SE}}_{A} = - 10\log \left( {\frac{{1 - S_{11}^{2} - S_{21}^{2} }}{{1 - S_{11}^{2} }}} \right)$$

where $P_{i}$ ($E_{i}$ or $H_{i}$) and $P_{t}$ ($E_{t}$ or $H_{t}$) are the power (intensity of the electric or magnetic field) of the incident and transmitted waves, respectively. *S*_11_ and *S*_21_ are the scattering parameters of the forward reflection and backward transmission coefficients which measured values, respectively.

Normally, ${\text{SE}}_{M}$ is considered more important to electrically thin materials. The $SE_{M}$ calculated as Eq. (14):

14 $${\text{SE}}_{M } = 20\log_{10} \left( {1 - e^{{\frac{ - 2t}{\delta }}} } \right) = 20\log_{10} \left( {1 - 10^{{\frac{{{\text{SE}}_{A} }}{10}}} } \right)$$

where $t$ is the thickness of the shielding materials, $\delta$ is the skin depth. The ${\text{SE}}_{M}$ effect can be safely neglected (${\text{SE}}_{M} \approx 0$) when ${\text{SE}}_{A}$ is higher than 10 dB since the amplitude of the EM waves first reaching the second interface is negligible. The multiply reflected EM energy loses its strength eventually via reflection and absorption [58]. Thus, ${\text{SE}}_{{\text{Total }}}$ can be expressed as Eq. (15):

15 $${\text{SE}}_{{\text{Total }}} = {\text{SE}}_{A} + {\text{SE}}_{R} .$$

The data showed that SE_*A*_ improved with the increasing loading amounts of the SWCNTs up to 2% by weight and then decreased slightly, as shown in Fig. 5b. However, *SE*_*R*_ exhibited a trend opposite to that of *SE*_*A*_, which means that the absorption behaviors become predominant with the increasing SWCNT loading amounts while the reflection decreases. This indicates that the occurrence of undesirable reflection caused by secondary pollution was diminished by the presence of the SWCNTs [59].

It is well documented that a mismatch of the electrical conductivities between nonconductive polymers and conductive fillers results in polarization and/or charge accumulation at the interfaces, leading to an increase in the dielectric loss. The loss of EM waves via absorption is mainly responsible for the CPCs due to their multiple interfaces arising from the polymer–nanofillers and nanofillers–nanofillers [60]. In this study, we used epoxy as a matrix to incorporate the SWCNT/rGO nanofillers that interact with the EM waves. In addition, epoxy can assist in suppressing the EM waves via absorption, reflection, or multiple reflections. The hybrid SWCNT/rGO nanofillers provided the impedance mismatch at the various interfaces, leading to enhanced interfacial polarization loss and multiple scattering in the epoxy matrix. These lead to an increase in the high dielectric loss for the absorption-dominant EMI-shielding [61].

To further explore the EMI-shielding mechanisms of the composites, we determined the EMI-shielding coefficient values of three contributions, *i.e*., absorption ($A$), reflection ($R$), and transmission ($T$), from the scattering parameters using the following Eqs. (16)–(18) [62, 63]:

16 $$1 = A + R + T$$

17 $$R = \left| {\frac{{E_{r} }}{{E_{i} }}} \right| = \left| {S_{11} } \right|^{2} = \left| {S_{22} } \right|^{2}$$

18 $$T = \left| {\frac{{E_{t} }}{{E_{i} }}} \right| = \left| {S_{21} } \right|^{2} = \left| {S_{12} } \right|^{2}$$

where $A$, $R$, and $T$ are coefficients of absorption, reflection, and transmission, respectively. $S_{11}$, $S_{22}$, $S_{21}$, and $S_{12}$ are the scattering parameters obtained from the measurements, and $E_{i}$,$E_{r} ,$ and $E_{t}$ are the intensities of the incident, reflected, and transmitted wave, respectively.

Most incoming EM waves were suppressed by absorption and reflection, while transmission was negligible in the S-$x$/G/PF/Ep composites (Fig. 5c). In G/PF/Ep, the $A$ and $R$ values of 0.37 and 0.64, respectively, were obtained, indicating the reflection-dominant EMI-shielding characteristics. This could be explained by the well-stacked laminar structures of the rGO nanosheets on the PF surfaces that serve as a reflective mirror for the EM waves [64]. However, $A$ was observed to increase sharply with the increasing SWCNT amount up to 2% by weight and then decreased, and vice versa was observed for $R$*.* This clearly indicates that the absorption-dominant behaviors become predominant with the presence of the SWCNTs. As expected, the addition of the SWCNTs plays important roles in establishing an electron percolation network and improving the interfacial properties between the SWCNTs/rGO and the epoxy matrix. The hierarchical structures of the SWCNTs/rGO led to an increase in multiple interfaces, resulting in the absorption-dominant characteristics of the EM waves. It was also confirmed that the excessive loading of the SWCNTs lowers the contribution to the absorption mechanism due to the agglomeration of the SWCNTs.

**Fig. 6:**
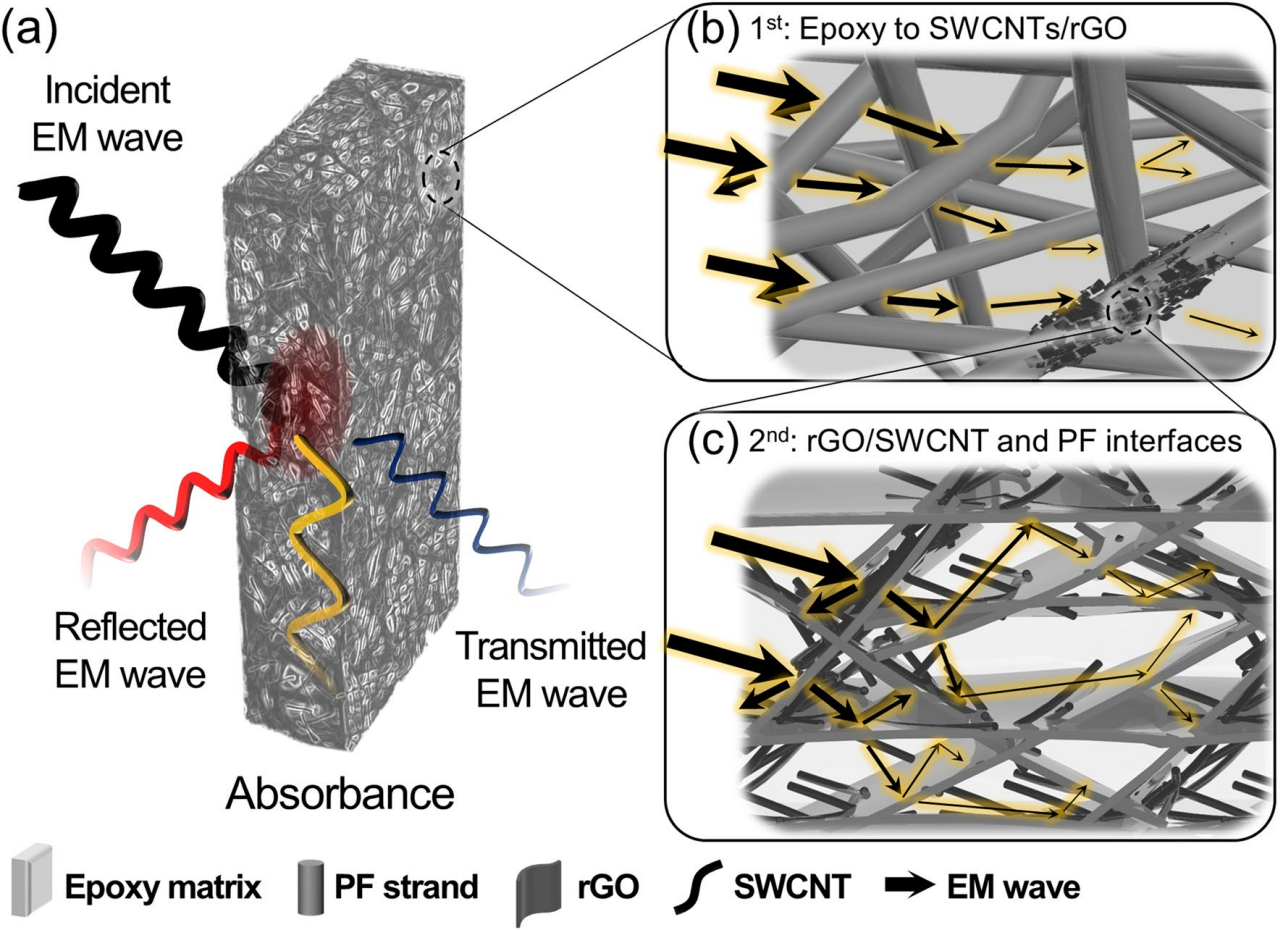
Schematic illustrations of the EMI-shielding mechanisms for the carbon ink-loaded PF/epoxy composites: **a** incident wave dissipations through S-*x*/G/PF/Ep, **b** absorption-dominant mechanism in the hierarchical structures of the porous PF structures, and **c** multiple scatterings of the EM waves at the interfaces between the SWCNTs/rGO and epoxy

Figure 6 shows the EMI-shielding mechanisms of the carbon ink-loaded PF/epoxy composites. The incident waves entering the EMI-shielding materials are attenuated by reflection and absorption. The shielding in the composites is certainly due to the high conductivity of the layer owing to the presence of the hybrid SWCNTs/rGO fillers. Meanwhile, another possible mechanism, which is beneficial for good shielding performance, is that the permeated EM waves within the composites can be drastically attenuated by passing through a polymeric matrix to SWCNTs/rGO; the dielectric loss results in a mismatch of the electrical conductivities at the interfaces of the polymer–nanofillers and nanofillers–nanofillers. Thus, it confirmed that the optimized SWCNTs/rGO networks facilitate multiple scattering due to the hierarchical structures, providing additional interfaces and thereby improving the interfacial properties. Then, the randomly oriented porous structures of the PFs provide effective dissipation of the incoming EM waves. The EM waves are secondarily attenuated at the interface of the SWCNTs/rGO and PF substrates [65, 66]. A similar study regarding the effect of hierarchical structure for multi-dissipative EMI-shielding was reported. It appeared that the 3D hierarchical network from *in situ* grown CNT on SiO_2_-coated carbon foam provided the efficient EMI attenuation while avoiding the secondary EM waves. The unique structure facilitates the absorption-dominant mechanism, effectively confining the EM waves and thereby attenuating the EM energy by internal reflection [66].

We identified the EMI-shielding performances of electrically conductive nanofillers-loaded polymeric composites (CPCs), as summarized in Fig. 5d [21, 30, 67–77]. Owing to efficient EMI-shielding performances, the CPCs exhibit advantages of lightweight, high flexibility, and ease of processing with the benefit of low cost [78]. The CPCs containing carbonaceous nanomaterials are known to absorb EM waves, leading to an absorption-dominant EMI-shielding mechanism. Normally, the EMI-*SE* increases with the increasing thickness of the EMI-shielding materials [79]. However, these would not be desirable for the high-performance EMI-*SE* in portable, wearable, and miniaturized electronics. Because the thicker the materials, the less flexible they are and the less dispersible the nanofillers are. To this end, we compared the EMI-shielding performance-to-thickness (EMI-*SE*/*t*, denoted as “*η*”) to compare the efficiency, as listed in Table 3. We found that our sample of S-2/G/PF/Ep exhibited the highest *η* value of 66.8 dB mm^–1^ with a 0.6 mm thickness.

**Table 3:** EMI-shielding performances at 8.2–12.4 GHz frequency of various electrically conductive nanofiller-loaded polymeric composites

Conductive fillers	Matrix	Thickness mm)	EMI-SE (dB)	*η* ^a^ (dB mm^–1^)	Dominant characteristic ^b^	Refs.
SWCNT/rGO (S-2/G/PF/Ep)	Epoxy	0.6	40.1	66.8	*A*	This study
MWCNT sponge	Epoxy	2.0	40.0	20.0	*A*	[67]
MWCNT/rGO	PDMS ^c^	2.0	31.2	15.6	*A*	[21]
MWCNT/rGO	PMMA^d^	2.0	43.0	21.5	*A*	[30]
rGO	PC ^e^	2.0	15.0	7.5	*A*	[68]
rGO	Epoxy	0.8	27.0	26.3	*A*	[69]
rGO aerogel	Epoxy	4.0	25.0	6.3	*A*	[70]
rGO/Fe_3_O_4_	PPy ^f^	2.5	48.6	19.2	*A*	[71]
MXene	PVA ^g^	0.3	21.0	70.0	*R*	[72]
MXene	PU ^h^	0.3	30.0	90.0	*R*	[73]
MXene	HEC ^i^	0.1	22.0	220.0	*R*	[74]
MXene	PS ^j^	2.0	62.0	31.0	*A*	[75]
MXene	Epoxy	2.0	41.0	20.5	*A*	[76]
MXene	Aramid fabric	1.0	35.7	35.7	*R*	[77]

^a^Efficiency of EMI-*SE* to thickness

^b^Dominant EMI-shielding mechanism: reflection (*R*) or absorption (*A*) characteristics from literatures

^c^Polydimethylsiloxane

^d^Poly(methyl methacrylate)

^e^Polycarbonate

^f^Polypyrrole

^g^Poly(vinyl alcohol)

^h^Polyurethane

^i^Hydroxyethyl cellulose

^j^Polystyrene

Specifically, MXene-loaded CPCs showed relatively high shielding performances due to their high electrical conductivities, in which abundant free electrons directly interact with the incident EM waves, leading to the high EMI-shielding by the reflection-dominant mechanism. Considering that the reflection-dominant mechanism gives rise to secondary or repeated EM pollution issues, the absorption-dominant EMI-shielding materials are more desirable. Besides, most of the research on the CPCs has focused on thermoplastic resins, including polyurethane (PU), poly(methyl methacrylate) (PMMA), and polycarbonate (PC), to improve the EMI-shielding performance with the benefits of recyclability, ease of processability, and shorter fabrication time. However, the carbon ink-loaded PF/epoxy composites were prepared by a facile dipping method. They exhibited superior mechanical durability with high flexibility and high EMI-shielding performance resulting from the enhanced interfacial interactions between the nanofillers (SWCNTs/rGO) and the epoxy [80]. We believe that epoxy as a thermosetting polymer would be a good alternative for EMI-shielding materials.

## Conclusions

In this work, we proposed carbon ink-loaded PF/epoxy composites for ultrathin, robust, and absorption-dominant EMI-shielding materials fabricated by a facile dipping method. This strategy successfully overcame the brittleness of a robust thermosetting matrix (epoxy) using PF and demonstrated high EMI-*SE* and superior mechanical durability. The highest EMI-*SE* was found to be ~ 40.1 dB in S-2/G/PF/Ep, with a thickness of 0.6 mm (66.8 dB mm^−1^) at 8.2–12.4 GHz and with the absorption-dominant EMI-shielding behaviors (absorption coefficient of 0.7). These absorption-dominant behaviors can be attributed to the effective dielectric loss at multiple interfaces and enhanced dissipation of the EM waves by the prolonged EM pathway in hybrid SWCNT/rGO networks, regardless of the thickness. Further, we also observed a strong correlation between the interfacial behaviors and the EMI-*SE*. The interfacial interactions of the composites play an important role in enhancing the EMI-*SE*. The multiscale porous structures, composed of 3D microscale pores of the SWCNT/rGO hybrids and macrovoids of the PF skeleton, contributed to enhancing the interfacial interactions. This facilitated the increase in impedance mismatch, interfacial polarization loss, and multiple scattering within the composites for efficient EMI-shielding performance. Therefore, we believe that this approach provides hybrid SWCNTs/rGO/PF/epoxy composites that could be used as highly flexible and robust EMI-shielding materials and hold great promise to meet the high demand for portable and wearable electronics.
